# Beyond clocks: A staged approach to classifying atrial fibrillation

**DOI:** 10.1016/j.hrcr.2024.06.001

**Published:** 2024-07-15

**Authors:** Margaret Harvey

**Affiliations:** Department of Acute and Tertiary Care, College of Nursing, University of Tennessee Health Science Center, Memphis, Tennessee

## Introduction

Atrial fibrillation (AF) is the most common arrhythmia worldwide and has an anticipated prevalence of 12.1 million in the United States alone by 2030.^1^ AF is associated with significant morbidity and mortality, with an estimated 28.4 billion US dollars being spent on health care in 2016.^1^ According to the 2023 ACC/AHA/ACCP/HRS Guideline for the Diagnosis and Management of Atrial Fibrillation,^2^ AF is now classified according to its stages versus its duration. The current classification system is based on the length of time a patient has been in AF, with an emphasis on therapeutic interventions, and is classified as paroxysmal, persistent, long-term persistent, or permanent.^2^ Terms like “valvular and nonvalvular,” “lone,” and “chronic” are obsolete and no longer used. This new classification system accounts for the progressive nature of the disease, with an increased emphasis on prevention, screening, and risk factor modification, with tailored strategies being based on the AF stage.

## New staged classification

The new AF classification system builds upon current nomenclature and includes 2 new categories, “At Risk” and Pre-AF,” that emphasize prevention, screening, and risk factor modification, for a total of 4 stages.^2^ Stage 1, “At Risk,” includes modifiable risk factors, such as obesity, diabetes, hypertension, and alcohol, and nonmodifiable risk factors, such as genetics, male sex, and age. Stage 2, “Pre AF,” encompasses patients with evidence of structural or electrical findings predisposing to the development of AF, such as atrial enlargement, frequent atrial ectopy, and atrial flutter. Stage 3, “AF,” includes patients with a diagnosis of AF who may transition into 1 of 4 substages: 3a, “Paroxysmal AF,” where AF is intermittent and terminates ≤7 days of onset; 3b, “Persistent AF,” where AF is continuous, sustains ≥7 days, and requires intervention; 3c, “Longstanding Persistent,” where AF is continuous for ≥12-month duration; and 3d, “Successful AF Ablation,” with freedom from AF after successful percutaneous or surgical intervention to eliminate AF.^2^ The last stage, 4, represents “Permanent AF,” where no further attempts are made at rhythm control, and is a shared decision with the patient and clinician.^2^

The new classification system also guides management based on stages while recognizing patients may transition among different substages of stage 3. The new guidelines also recommend providers address modifiable risk factors regardless of stage, increase AF surveillance for stage 2, and continue monitoring for AF burden in stage 3. For patients in stages 3 and 4, it is important to determine if AF is associated with pathologic changes, conduct a stroke risk assessment with oral anticoagulants as indicated, and treat symptoms. The new AF classification system also highlights the role multiple stakeholders have in the care of patients with AF, emphasizing the need for holistic and comprehensive care during the entire lifespan of an individual. Using a stage-based approach versus duration of AF also emphasizes the role of shared decision-making and uses a more tailored approach to treating AF.^2^

## Conclusion

It is no longer acceptable to treat AF based solely on how long a person has had the condition. Current evidence suggests adopting a more holistic approach that includes risk factor modification, primary prevention, behavior modification, and screening. The role modifiable risk factors play in the development of AF has become increasingly evident. Improved technologies in ambulatory cardiac monitoring and wearables also provide enhanced screening for AF in high-risk patients. The new stage-based approach to classifying AF reflects a paradigm shift from traditional treatment to an emphasis on prevention and screening. This approach requires multiple stakeholders, including primary care providers, who also play a pivotal role in providing a tailored approach to AF prevention and management.

## Disclosures

The author has no conflicts of interest to disclose.

## References

[1] January C.T., Wann L.S., Alpert J.S. (2014). 2014 AHA/ACC/HRS guideline for the management of patients with atrial fibrillation: a report of the American College of Cardiology/American Heart Association Task Force on Practice Guidelines and the Heart Rhythm Society. J Am Coll Cardiol.

[2] Joglar J.A., Chung M.K., Armbruster A.L. (2024). 2023 ACC/AHA/ACCP/HRS guideline for the diagnosis and management of atrial fibrillation: a report of the American College of Cardiology/American Heart Association Joint Committee on Clinical Practice Guidelines. Circulation.

